# *Primum non nocere*—first do no harm. And then feed peanut

**DOI:** 10.1186/s13223-017-0180-2

**Published:** 2017-02-08

**Authors:** Kyla Jade Hildebrand, Elissa Michele Abrams, Timothy K. Vander Leek, Julia Elizabeth Mainwaring Upton, Douglas P. Mack, Linda Kirste, Christine McCusker, Sandeep Kapur

**Affiliations:** 1 Program Director Clinical Immunology & Allergy Training Program, Faculty of Medicine, Department of Pediatrics, University of British Columbia, British Columbia Children’s Hospital, 4480 Oak Street, Room 1C31B, Vancouver, BC V6H 3V4 Canada; 2 University of Manitoba, FE125-685 William Ave, Winnipeg, MB R3E 1B2 Canada; 3 University of Alberta, 207-10430 61 Ave NW, Edmonton, AB T6H 2J3 Canada; 4 University of Toronto, the Hospital for Sick Children, 555 University Avenue, Toronto, ON M5G 1X8 Canada; 5 McMaster University, Joseph Brant Hospital, 1230 North Shore Blvd, Burlington, ON L7S 1W7 Canada; 6 Government of British Columbia, Dietitian & Physical Activity Services, HealthLink BC, Burnaby, Canada; 7 McGill University, Meakins-Christie Laboratories RI-MUHC, Block E, Office EM3.2230,2155 Decarie Blvd, Montreal, QC H4A 3J1 Canada; 8 Dalhousie University, 503-5657 Spring Garden Road, Halifax, NS B3J 3R4 Canada

**Keywords:** Peanut allergy, Prevention, High-risk, Infant

## Abstract

The *Addendum Guidelines for the Prevention of Peanut Allergy in the United States*—*Report of the NIAID*-*Sponsored Expert Panel* were developed to build on previous food allergy guidelines after several key studies demonstrated the benefit of early introduction of allergenic foods. These landmark studies including the Learning Early about Peanut (LEAP), LEAP-On and Enquiring about Tolerance trials created a paradigm shift in food allergy prevention. The “take home” messages of this guideline include that peanut should be introduced early in the first year of life, and for the majority of infants, peanut can be introduced at home. The only group of infants for which medical assessment is recommended is those with severe eczema, egg allergy or both. Here we summarize the Guideline recommendations, endorsed by the Canadian Society of Allergy and Clinical Immunology, and highlight important aspects relevant to Canadian practitioners.

The *Addendum Guidelines for the Prevention of Peanut Allergy in the United States*—*Report of the NIAID*-*Sponsored Expert Panel* were developed to build on previous food allergy guidelines after several key studies demonstrated the benefit of early introduction of allergenic foods [1, 2]. These landmark studies including the Learning Early about Peanut (LEAP) [3], LEAP-On [4] and Enquiring about Tolerance [5] trials created a paradigm shift in food allergy prevention. We commend the authors of the Guidelines for recognizing the need for prompt dissemination of the findings. Here we summarize the Guideline recommendations, endorsed by the Canadian Society of Allergy and Clinical Immunology (CSACI), and highlight important aspects relevant to Canadian practitioners.

The Guidelines address the prevention of peanut allergy among three groups of infants. The “take home” messages include that peanut should be introduced early in the first year of life, and for the majority of infants, peanut can be introduced at home. The only group of infants for which medical assessment is recommended is those with severe eczema, egg allergy or both. In this group, the Guidelines suggest skin prick testing and/or peanut-specific IgE evaluation prior to peanut introduction around 4–6 months of age. Recognizing that timely access to subspecialist allergists can be limited, the Guidelines suggest that non-allergy physicians may consider performing a peanut-specific IgE level as an initial step for infants at high risk of peanut allergy. Testing for food allergy by non-allergy physicians, the authors wrote, has the potential to reduce the number of infants needing allergist screening by supporting home introduction. However, this recommendation warrants further discussion.

The definition of severe eczema is intended to classify patients who continue to experience frequent and extensive symptoms despite *optimal* management and adherence to treatment. However, it is our experience that many parents and healthcare providers use the term “severe” to refer to any patient presenting with bothersome symptoms, regardless of treatment. This discrepancy could lead to a significant increase in infants with mild or sub-optimally managed eczema deemed inappropriately as high risk for peanut allergy. Many infants could undergo unnecessary testing, thereby missing the window of opportunity of early peanut introduction. In the absence of specific IgE mediated symptoms, a positive skin/food-specific IgE test represents sensitization however does not prove clinical reactivity to the food. Individuals with atopic dermatitis, or other allergic conditions, are more likely to have elevated IgE levels, and are more likely to have false positive food-specific IgE tests [6].

Additionally, by recommending that non-allergy physicians perform peanut-specific IgE to help facilitate timely assessment of infants at high risk, our concern is that the opposite may result: referrals to subspecialty allergists may increase for assessment of false positive sIgE results among sensitized individuals. The increased wait time for allergy assessment may lead to further unnecessary delay in the introduction of peanut and possibly other foods. The Guideline authors emphasize that an undetectable peanut-specific IgE level has a “strong negative predictive value”. However, many infants with a personal and/or family history of atopy will have clinically irrelevant sensitization identified by this test. The Guideline authors recommend that an infant with a detectable peanut-specific IgE level “be referred to a specialist for further consultation”. It is our concern that many of these infants will instead continue to strictly avoid peanut and will not seek further assessment by a subspecialty allergist or be unable to see an allergist in a timely fashion.

Another concern is that healthcare providers less familiar with the pitfalls of ordering sIgE tests may order testing to foods other than peanut, even though the Guidelines specifically discourage this practice. A recent study determined that in an unselected population, food allergy panel testing had a positive predictive value of only 2.2% [7]. Similarly, many laboratories that process requisitions for peanut-sIgE automatically substitute a food ‘mix’ test. While a negative food ‘mix’ test would reasonably rule out clinically relevant peanut sensitization, a positive test does not identify which food from the mix to which an individual is sensitized and would result in testing for 5–6 additional foods. Each additional food yielding a positive result would necessitate further evaluation and potential delayed introduction.

Finally, care must be taken to ensure feeding infants first foods is not a medical act. An observational study found a low prevalence of peanut allergy in settings in which normal feeding practices included peanut among an infant’s first solid foods [8]. As per the LEAP protocol, the Guidelines recommend that infants who tolerate peanut should continue to consume 6–7 grams over 3 servings each week. It is essential to make a distinction between what is feasible in a research setting and that which is practical and appropriate in the home setting.

How should one interpret the Guidelines and apply them to practice? Our recommendations include the following:

- The overwhelming majority of infants, including those with mild to moderate eczema, can introduce peanut early and at home without investigation.
- Early introduction of peanut is the primary goal as it is evident that there is an early window of opportunity for the development of tolerance.
- Peanut-specific IgE testing by non-allergist physicians should be considered for “at risk” infants only when a referral to an allergist is not available in a timely manner.
- Testing for foods beyond peanut, or the use of food panels, with specific IgE testing is strongly discouraged. Education of non-allergist physicians on the pitfalls of specific IgE testing is necessary in order to reduce harm.
- Subspecialty allergists have a duty to provide infants at high risk for peanut allergy timely access to consultation early in their first year of life, and to offer in clinic, observed first ingestion of peanut, when needed.

The increase in food allergy prevalence in recent decades is a public health problem and may in part be due to years of recommending delayed introduction of foods based on expert opinion only. We thank the authors of this Guideline for their collaboration in creating this timely document. Bearing in mind the issues discussed in this editorial, it is our hope that a strong message is heard that early introduction of peanut is the goal for most infants. First do no harm—and then feed peanut.
